# Towards Improving the Outcomes of Assisted Reproductive Technologies of Cattle and Sheep, with Particular Focus on Recipient Management

**DOI:** 10.3390/ani10020293

**Published:** 2020-02-13

**Authors:** Jamee Daly, Hayley Smith, Hayley A. McGrice, Karen L. Kind, William H.E.J. van Wettere

**Affiliations:** School of Animal and Veterinary Sciences, University of Adelaide, Roseworthy, SA 5371, Australia; a1668187@student.adelaide.edu.au (H.S.); hayley.mcgrice@adelaide.edu.au (H.A.M.); karen.kind@adelaide.edu.au (K.L.K.);

**Keywords:** recipient management, recipient animal, anti-Müllerian hormone, AMH, assisted reproductive technology, ART, sheep, cow

## Abstract

**Simple Summary:**

The Australian livestock industry has grown significantly over the last decade. In order to meet national consumer demands, as well as the growing export market, more efficient means of producing lamb and beef products are required. Assisted reproductive technologies (ARTs) can be used to increase genetic gain and improve overall herd reproductive potential. This review focuses particularly on the management and selection of recipient animals utilised in ARTs. The condition and quality of the recipient animal is pivotal to the efficiency of reproductive technologies, as the inability of an embryo to establish and maintain pregnancy is the most significant cause of reproductive losses. A variety of external, uncontrollable factors affect the reproductive potential of an individual within any given reproductive season, limiting reproductive efficiency. Therefore, improved selection and management of recipient animals can help to increase the productivity of the Australian livestock industries.

**Abstract:**

The Australian agricultural industry contributes AUD 47 billion to the Australian economy, and Australia is the world’s largest exporter of sheep meat and the third largest for beef. Within Australia, sheep meat consumption continues to rise, with beef consumption being amongst the highest in the world; therefore, efficient strategies to increase herd/flock size are integral to the success of these industries. Reproductive management is crucial to increasing the efficiency of Australian breeding programs. The use of assisted reproductive technologies (ARTs) has the potential to increase efficiency significantly. The implementation of multiple ovulation and embryo transfer (MOET) and juvenile in vitro fertilization and embryo transfer (JIVET) in combination with genomic selection and natural mating and AI is the most efficient way to increase genetic gain, and thus increase reproductive efficiency within the Australian livestock industries. However, ARTs are costly, and high variation, particularly between embryo transfer recipients in their ability to maintain pregnancy, is a significant constraint to the widespread commercial adoption of ARTs. The use of a phenotypic marker for the selection of recipients, as well as the better management of recipient animals, may be an efficient and cost-effective means to increase the productivity of the Australian livestock industry.

## 1. Introduction

Commensurate with population growth, there has been a 58% increase in global meat consumption over the last 20 years, with 4% of this growth attributed to increases in per person consumption [1]. Beef and veal accounted for 21% of this increase and sheep meat accounted for 5%. The Australian agricultural industry is one of the largest contributors to Australia’s economy, constituting 3% of the total gross domestic product (AUD 47.7 billion) [2]. Australia was the world’s largest exporter of sheep meat and the third largest exporter of beef, behind Brazil and India in 2017. In the past 20 years, the global consumption of sheep meat has increased by 64% and is projected to continue to increase by 1.8% in the next decade [1]. Although Australians consume roughly double the global average of sheep meat, the Australian sheep flock makes up only 7% of the world’s domestic sheep population [3]. At 26 kg per person per year, Australian beef consumption is amongst the highest in the world; however, the Australian beef herd make up less than 3% of the world’s domestic beef population [4]. Domestic consumption has a gross value of AUD 11.4 billion for the Australian cattle and calf production industry, and yet Australia imported AUD 24.7 billion [5] in food products, which may be due to economic factors including the high Australian dollar, and a shift in supply domestically, as well as national and international competition [6]. Alternatively, the increasing consumer demand for these products and an inability to produce the food efficiently within our country could be a contributing factor. Together, this demonstrates the need to produce more red meat within Australia. 

Pure Merino flocks make up almost 55% of the Australian sheep flock, with the remaining 45% including Merino cross, dual purpose and other breeds [7]. Beef breeds are more varied, with both *Bos indicus* and *Bos Taurus* being utilised depending on the climate across the country [8]. Holsteins and Jerseys are common dairy breeds in Australia [9]. Generally, ewes are mated naturally or through artificial insemination (AI). Due to ongoing drought conditions, there is an imperative for most Australian farmers to increase retention rates of female lambs with the aim of increasing breeding flock size and reproductive efficiency [10]. Assisted reproductive technologies are an effective and efficient strategy to improve offspring numbers within the Australian flock. Multiple ovulation and embryo transfer (MOET) and both mature and juvenile in vitro fertilisation and embryo transfer (MIVET/JIVET) can increase the number of offspring per mating from desirable animals, therefore increasing genetic gain [11]. JIVET also reduces the generation interval significantly, as oocytes can be harvested from lambs as young as six weeks old. The success of these programs, as with natural mating and AI, relies on reproductively fit animals. Currently, the selection of all breeding ewes is based on maternal reproductive performance, along with the reproductive performance evaluation of the ewe over her lifetime. Granleese et al. [12] reviewed the value of using genomic selection, AI or natural mating and MOET/JIVET to the Australian sheep industry. The use of MOET with either natural mating or AI yielded an extra 30% in genetic gain. This genetic gain was further increased when used in combination with genomic selection, and again with the addition of JIVET (21% increase). These data demonstrate the value of combining genomic selection and ARTs to the Australian sheep industry [12]. Unfortunately, there is large variation between individuals in reproductive parameters such as the ovarian response to stimulation with reproductive hormones, the number of embryos recovered, and pregnancy and lambing rates [13]. These outcomes are greatly affected by external stressors such as feed availability and climate, as well as the ART implemented. The increase in genetic gain is costly, and there is a need to select animals for inclusion in ARTs, either as oocyte/embryo donors or embryo recipients, which perform well, even when environmental conditions are unfavourable.

As such, reliance on the inheritance of reproductive traits is difficult, and current variation in reproductive performance of the Australian sheep flock demonstrates that additional selection methods are required. In the current market, a phenotypic marker of reproductive selection would be ideal, as it could be implemented across varying breeding enterprises. The primary focus of studies investigating strategies to improve the outcomes of ART has been on improving superovulation protocols, as well as the quality of oocytes and embryos produced. However, the sub-optimal selection of embryo recipients contributes significantly to the outcomes of ART [14] and yet has received less attention in the literature. Therefore, the focus of this review is the selection and management of embryo recipient animals for the optimisation of pregnancy establishment and maintenance. To that end, this review will focus on three key points: (1) factors that influence the efficiency of embryo transfer-based assisted reproductive technologies (2) the determinants of pregnancy establishment and maintenance, and (3) strategies to improve pregnancy rates through improved recipient selection, management and the use of interventions.

## 2. Assisted Reproductive Technologies

As the industry moves to increase the number of lambs produced, and increase the size of the national ewe flock, the effective implementation of assisted reproductive technologies becomes increasingly important. From the second half of the 1980s, in vitro production of embryos (IVP) became a widely used technology within livestock industries [15]. IVP involves three main procedures, initiated from the collection of immature oocytes from the ovary, from either stimulated or unstimulated animals. Ultimately, the aim of the development and implementation of these protocols is to increase the rate of genetic gain within Australian livestock industries, thereby increasing flock numbers. The oocytes undergo in vitro maturation (IVM), in vitro fertilisation (IVF) and in vitro culture (IVC) [15]. This generates an in vitro produced embryo which is then transferred into a recipient animal [15]. Embryo transfer (ET), MOET, and IVP from either mature (MIVET), or juvenile animals (JIVET) are the key ARTs under investigation. MOET is a conventional embryo flush commonly used in the cattle industry and consists of stimulation with reproductive hormones to induce the release of multiple oocytes from donor cows, followed by insemination, as opposed to the release of one oocyte during a natural oestrus. Essentially, MOET increases the reproductive rate of a donor, with multiple embryos being flushed and placed directly into recipients. In comparison, JIVET involves the superovulation of donor lambs or calves at two to three or five to six months of age, respectively, and the harvesting of their oocytes followed by in vitro embryo production and embryo transfer into mature recipients [11,16]; this same process can also be undertaken in mature donor ewes and cows (MIVET). It is evident that male reproductive performance is more heritable than female reproductive performance [17], and this is facilitated through the widespread adoption of AI. However, MOET, MIVET and JIVET provide an effective strategy to increase the use of superior male and female genetics, further enhancing the rate of genetic gain.

Within each ART, multiple strategies exist to stimulate and synchronise the oestrus of the donor and recipient [18], causing large variation in individual responses. The majority of these protocols involve the use of prostaglandin F2 alpha (PGF2α) to induce luteolysis, and slow release progesterone implants (i.e., controlled internal drug release devices- CIDRs) to synchronise oestrus. The use of a CIDR delays the onset of ovulation and induces cyclicity in non-cycling recipients, resulting in synchronised ovulation upon removal [19]. The use of exogenous hormone protocols to synchronise the timing of oestrus and ovulation, and increase progesterone production in recipients, is essential for the success of ET programs with various combinations of exogenous gonadotrophins used to stimulate follicle growth and ovulation (reviewed by Abecia, Forcada and Gonzalez-Bulnes [18]). The combination of hormones used, the timing and duration of treatment with these hormones, and the doses used, can all vary within a stimulation or synchronisation protocol [20,21,22,23]. Furthermore, the type of intravaginal device [24], as well as the origin of the hormone (synthetic or animal or human derived) [25], can vary. The relative merits of the large number of protocols reported in the literature for synchronising oestrus and stimulating ovulation have been reviewed previously [20,26,27,28,29,30,31,32,33,34] and will not be discussed here. However, it is well accepted that the efficacy with which these protocols synchronise and stimulate ovulation varies, with further variation resulting from phenotypic differences between the animals involved.

It is common for high incidences of embryo loss to limit reproductive efficiency in natural mating systems, and these can be extrapolated to affect outcomes of ARTs. In ewes, most pregnancy losses occur in the first month of pregnancy, with pregnancy rates at day 30 being between 40% and 60% [35]. In naturally mated Merino ewes, 42% of potential lamb losses occur within 20 days of mating in South Australian flocks (59.7 ova lost per 100 ewes) [36], with losses of 52.6% reported for Katandin ewes [22]. Similarly, following AI of North Patagonia Merino ewes, 7.2% (7/97) of ewes were not pregnant 33 days later, with only a 1% (1/97) loss observed between days 33 and 90 post-AI [37]. It is, therefore, apparent that high incidences of embryo loss limit reproductive efficiency in naturally mated and artificially inseminated sheep. Specifically, it is the quality of the maternal hormonal and uterine environment which allows for successful pregnancy establishment. Further to this, embryonic mortality following embryo transfer is a persistent problem in cattle. Comparing calving rates from multiple ET programs across 9 years, it was concluded that up to 47% of embryo mortality occurs between the first and second trimester [38,39]. This is important, as Peterson and Lee [38] outlined a far greater pregnancy loss in cattle that are embryo recipients as opposed to those that are artificially inseminated. Similarly, a pregnancy rate of 58% was found in embryo recipient ewes, compared with 75% in the naturally mated group [40].

In sheep, 25% to 50% of embryonic losses occur during the early embryonic period, with possible failure resulting from luteal insufficiency, failure to supply progesterone to the uterus and failure of maternal recognition of pregnancy [41]. Similarly, in cattle, a meta-analysis of pregnancy loss in beef cattle determined that pregnancy loss during early embryonic development was 47.9% [42]. When broken down further, 28.4% of losses occurred by Day 7, 3.9% between Days 7 and 16, and 15.6% from Day 16 to Day 32 [42]. Similar to sheep, only 5.8% of pregnancy loss was accounted for by late embryo and early fetal losses [42]. Pregnancy failure before or around the time of maternal recognition of pregnancy accounts for the majority of pregnancy losses in cattle [38,39,42]. Similarly, only 42% of IVP embryos survive by day 60 in recipient ewes [40]. A model developed by McMillan [43] determined that variation in the ability of a recipient to carry a pregnancy to term, rather than embryo quality, was the major source of variation rates following ET. This suggests the early maternal environment plays a significant role in successful pregnancy. Understanding the synchrony in the timing of oestrus and post-ovulatory uterine changes between the donor and recipient is crucial, as they are likely to be determinants of pregnancy rates following embryo transfer. Pregnancy rates were 64% when cattle recipients expressed oestrus within six hours of the donor cow, with pregnancy rates dropping to 56% and 48% when recipients were in oestrus within 12 or 24 h, respectively, of the donor cow [44]. Not only does this demonstrate that there is an optimal environment for an embryo to survive (discussed in detail herein) it also demonstrates the importance of the synchrony of the recipient animals used in an assisted reproductive technology.

## 3. Embryo Implantation and Pregnancy Establishment

The suitability of a recipient for ET can be attributed to the timing of oestrus expression relative to the donor and the presence of a functional CL capable of producing sufficient progesterone to support embryo development and implantation as well as pregnancy maintenance [45]. Importantly, in ruminants, progesterone also plays a key role in regulating the release of prostaglandins from the endometrium and subsequent regulation of the oestrous cycle [46].

### 3.1. Corpus Luteum, Progesterone, Interferon Tau and Pregnancy Maintenance

The Corpus Luteum (CL) is the key structure regulating pregnancy maintenance. In cattle, an increase in CL size increases the circulating concentration of progesterone, resulting in a more suitable environment for early embryo development, implantation and pregnancy maintenance [47]. However, for a CL to persist and continue to produce progesterone, pregnancy must be established. Progesterone regulates the embryo–maternal relationship by stimulating the endometrial glands to synthesise and secrete histotroph, which is essential for embryo development, migration and implantation [41]. High concentrations of progesterone in the immediate post conception period advance conceptus elongation, increase interferon tau (IFNT) secretion by the embryo and result in greater pregnancy rates in cattle and sheep [48]. IFNT is a crucial controlling factor for the establishment of pregnancy in ruminant species [49]. Secreted by hatched ovine and bovine blastocysts on Days 10–12 and 14–17, respectively, IFNT is integral for implantation and maternal recognition of pregnancy [49]. IFNT inhibits endometrial expression of oxytocin receptors and oestrogen receptor alpha, stabilises expression of progesterone receptors and prevents secretion of the luteolytic hormone PGF2α [49]. The receptivity of the uterus to the implanting embryo relies on this reduced expression of PGF2α, and the successful implantation of ovine and bovine embryos on Day 16 and 19, respectively, also relies on IFNT stimulated alterations in the endometrial transcriptome [49].

Progesterone also plays a crucial role at this early stage of pregnancy establishment [50]. Both embryo development and the ability of the conceptus to secrete INFT is related to progesterone concentration [51]. Sustained exposure to progesterone downregulates endometrial PGR expression as the luteal phase of the oestrous cycle progresses, such that animals with high progesterone levels are receptive to embryo implantation earlier than those with low levels of progesterone [52]. Progesterone may affect the production of factors by the endometrium that support or regulate conceptus elongation, as uterine exposure to elevated progesterone prior to embryo transfer resulted in an advanced conceptus elongation [53].

### 3.2. Recipient Selection Based on Progesterone or CL Function

The continued production of progesterone is the most significant factor in the maintenance of pregnancy, and high progesterone concentrations during the week following ovulation are associated with improved pregnancy outcomes in cattle [48]. Therefore, selecting recipient ewes or cows based on either progesterone levels or CL function is likely to increase the probability of implantation and pregnancy following embryo transfer. Successful pregnancies in cattle have been reported in recipients with progesterone concentrations ranging from 0.58 ng/mL to greater than 16.9 ng/mL on the day of ET [19]. However, previous evidence indicates that conception rates were lower in recipients with progesterone levels below 1 ng/mL compared with those with levels greater than 3 ng/mL [54]. Selecting recipients based on the presence of a CL is not sufficient to ensure pregnancy, with pregnancy occurring in only 60% of recipients with CLs at the time of transfer, and a significant proportion of these animals failing to maintain pregnancy to term [55]. Therefore, measures of CL function (i.e., size, blood perfusion), may be a more appropriate strategy for recipient selection. Recipients with larger CLs typically have higher progesterone concentration and hence a uterine environment which is more suitable for pregnancy establishment [44]. Cattle which fail to maintain pregnancy tend to have low plasma progesterone concentrations, which suggests that the CL is not functioning effectively to support pregnancy [54]. A CL of ten millimetres in diameter at the time of embryo transfer is considered an acceptable size for a functional CL in cattle; however, no studies have reported an exact correlation between CL size and pregnancy rate [45]. Circulating progesterone levels are related to the CL blood perfusion [48], and Doppler ultrasound measures of CL blood perfusion appears to be a promising strategy with which one can identify recipients with increased probability of maintaining pregnancy [56]. The selection of recipients based on measures of CL function and/or size following synchrony may be an effective strategy to improve pregnancy outcomes. However, predicting CL function based on previous oestrous cycle measures of CL function may be a more cost-effective strategy for recipient selection, as it would enable selection prior to the receipt of costly hormonal synchronisation protocols. Further intervention strategies aimed at increasing progesterone and CL function may also benefit the Australian livestock industries through increasing the recipient receptivity to embryos.

## 4. Anti-Müllerian Hormone

Anti-Müllerian hormone (AMH) is a 140-kDa glycoprotein dimer of the transforming growth factor (TGF)-β family, and in female ruminants it is expressed solely by the granulosa cells of growing ovarian follicles [57]. AMH expression is initially detected in secondary follicles and maintained in greater levels in pre-antral and early antral follicles, and decreases as follicle size increases [58]. In ruminants, the later stages of antral follicle growth are induced by surge-like secretion of FSH [59], and AMH moderates the growth of pre-antral and small antral follicles by decreasing their responsiveness to FSH, effectively controlling the recruitment of follicles in the pre-ovulatory pool [58,60]. In sheep, AMH is present in the follicular fluid and granulosa cells of small (<2.5 mm), gonadotrophin-responsive antral follicles, as well as medium (2.3–3.5 mm) and large (3.5 to 8 mm) gonadotrophin-dependent antral follicles, and decreases as follicle size increases. Importantly, AMH is not produced by atretic follicles [58], making it an accurate marker of the number of healthy, growing antral follicles present on the ovary.

### 4.1. AMH as a Tool to Select Donors and Recipients for Inclusion in ART Programs

In both cattle and sheep, plasma AMH concentrations prior to exogenous gonadotrophin protocols are positively correlated with the number of oocytes and embryos produced [61,62,63,64]. Within cohorts of donor heifers and cows enrolled in MOET or MIVET programs, those with high AMH produce more total embryos [59] and more transferrable embryos [65]. Furthermore, the percentage of embryos transferred to recipients that became viable calves, was higher for embryos collected from donors with higher AMH concentrations [64]. Similar relationships have been reported for donor ewe lambs enrolled in JIVET programs [66]. Circulating progesterone levels in vivo influence fertilisation rates and embryo quality parameters, with studies indicating that intra-follicular progesterone and AMH also affect fertility. In women, elevated levels of both AMH and progesterone in follicular fluid were associated with greater blastocyst numbers [67]. Specifically, six of the follicular fluid samples studied had an AMH level >15 pmol/L and progesterone >60 mg/mL and oocytes from these follicles progressed to the blastocyst stage [67]. Although the potential benefits of selecting donor ewes and cows based on AMH are clear, the impact of AMH on pregnancy outcomes has received less attention, and, therefore, the relevance of AMH as a way to select recipients with increased fertility requires more extensive research.

### 4.2. AMH as a Phenotypic Marker of Pregnancy Rates

Only a few studies have investigated the potential of AMH to identify pregnancy outcomes, and these have produced conflicting results. In dairy cows, low AMH levels were associated with lower pregnancy rates [68], with higher pregnancy rates observed in animals with high AMH levels eight days after insemination [69]. In cattle, it is well established that antral follicle count and AMH are positively correlated [70,71,72]. Interestingly, whilst antral follicle count is indicative of fertility outcomes in heifers, fertility was not correlated with AMH. In dairy cows, however, cows with a low vs high AMH have reduced fertility and shorter herd longevity [72].

To date, two studies have investigated the relationship between prepubertal circulating levels of AMH and fertility. It was determined that for both Sarda ewe lambs [73] and Rasa Aragonesa ewe lambs [74], animals with high AMH concentrations were more fertile at their first lambing. Furthermore, animals with a high AMH level were 82 days younger at first lambing, and had a resulting 0.17 extra lambings, and 0.38 extra lambs per ewe per year [75]. Given that AMH may reflect ovarian function and reserve, and possibly pregnancy outcomes, future work is required in two key areas. One, to determine if there is an optimal timing of sampling to best reflect the status of the ovarian environment for optimum embryo receptivity; two, to determine if AMH is a marker of CL function, as this may help to differentiate between embryo induced and CL induced improvements.

## 5. Management of Recipient Animals

### 5.1. Body Condition Score and Nutrition

As mentioned earlier, reproduction is highly sensitive to external factors and, therefore, the management of recipient animals is crucial to the success of assisted reproductive technologies. Body condition score on a scale of one (severely emaciated) to five (obese) is a successful quantitative approach to identify the ideal body condition at which ET yields the highest success rate. Looney et al. [45] reported that 633 cattle with a BCS of three had a pregnancy rate of 55%, whereas pregnancy rate in 230 cattle with a BCS of one was 44%. Ideally for cattle, a BCS of two and a half at calving and two at the time of ET is considered ideal in achieving the highest pregnancy success rates [45,76]. The control of body condition is achieved through the correct nutritional management of the animal, as nutritionally induced metabolic changes affect reproductive parameters. Progesterone production, uterine function and health, along with embryo survival, are all impacted as a result of nutritional management [77]. Recipient nutrition is generally considered more influential than donor nutrition in terms of ET success rate [45]. A negative energy balance at the time of ET or early pregnancy results in pregnancy termination due to insufficient nutrient supply to the developing embryo [77]. Straw based diets, mouldy grain and sudden changes in nutrient intake can negatively impact ruminal microbes and fermentation, with an associated effect on circulating metabolites and hormones [77]. Recipients should be placed on an allocated diet six weeks prior to ET and the diet maintained until eight weeks of gestation. This helps prevent early embryo mortality as a result of altered hormones due to change in diet [77].

Once pregnancy is established, the recipient diet can be altered in relation to energy requirements; however, any change should be gradual [76]. A daily weight gain of 250–350 g/day resulted in higher pregnancy rates in cattle, with growth rates in excess of 350 g/day reducing pregnancy rate, hence establishing the threshold of 350 g/day for optimal ET success in recipients [77]. Whilst de Brun and colleagues [41] observed no effect of donor nutritional status on pregnancy establishment following embryo transfer in sheep, the nutritional status of the recipient was found to be critical for successful embryo survival following transfer. Plasma non-esterified fatty acids (NEFAs) increased in undernourished ewes, compared with maintenance fed ewes, the increased lipid mobilisation resulted in an increased pregnancy failure of 35% in undernourished ewes, compared with 14% in maintenance fed ewes [41]. In the same study, undernutrition resulted in reduced oocyte developmental competence, lower intra-uterine progesterone levels and fewer progesterone receptors. 

Inappropriate recipient nutrition not only reduces pregnancy rates, but in pregnancies that are maintained, it is highly likely to impair the growth and development of the conceptus. Nutritional insufficiency during pregnancy, along with exposure to heat stress, psychological stress, disease and environmental pollutants, is known to result in progeny with reduced growth and reproductive potential [78,79,80,81].

### 5.2. Synchronisation and Intervention Strategies

Progesterone supplementation, usually through the use of controlled internal drug releasing devices (CIDR), can act to support ovarian function and synchronise recipient animals, creating a favourable environment for embryo survival. In terms of recipient management, the focus should be on elevating progesterone immediately post conception; as this has been shown to advance uterine receptivity of the embryo and conceptus elongation as well as increase INFT [82]. Additionally, for both sheep and cattle, close synchrony between the embryo and uterine environment of the recipient is crucial for pregnancy success [82]. Asynchrony by as little as 2 days is detrimental; the transfer of Day 7 embryos to Day 5 or 9 uteri resulted in retarded or advanced conceptus at Day 14 [83]. As previously discussed, there are a multitude of protocols available to synchronise and stimulate cattle and sheep for ART [18]. Ultimately, intervention strategies should aim to increase circulating progesterone immediately following ovulation, whilst synchronisation strategies should aim to synchronise the donor to the recipient as both play important roles in embryo survivability.

As reviewed by Looney and colleagues [45], the time of oestrus expression and the presence of a functional corpus luteum is crucial for an effective recipient animal. The use of an intravaginal progesterone releasing device, along with intramuscular administration of oestradiol benzoate (2.5 mg) and progesterone (50 mg) is an example of a protocol that enables the synchronisation of the emerging follicular wave [45,84], enabling more efficient use of the recipient animals, as embryo transfer can occur without oestrus detection. It is known that oestradiol benzoate is essential at the time of CIDR insertion for the induction of a new follicular wave; however, its importance at the time of CIDR removal is still unclear [85]. Controlling follicular wave development can be achieved through follicle ablation or hormonally by treatments of GnRH, PGF, oestradiol and progestogen/progesterone combinations [84,85]. Oestrus synchronisation protocols controlling both the follicular and luteal phases are most effective [84]. These methods reduce the variability caused by treating animals that are at different stages of the oestrous cycle, which provides a more favourable environment upon transfer of the embryos [84].

Intervention strategies, involving exogenous gonadotrophins and progesterone, can also be applied for recipients. Implementation of these interventions at, or prior to, embryo transfer aims to increase circulating progesterone concentrations, as this increases pregnancy rates (as discussed earlier). A significant amount of research has investigated altering the timing and dosing of CIDRs to achieve this [45]. Thompson, Bell, McMillan, Peterson and Tervit [40] placed ewe recipients into two groups; one group received a fresh CIDR post ET for eight days and the control group did not receive the additional CIDR. The administration of the additional CIDR post transfer did not result in an increase in pregnancy rate or embryo survival. Similarly, the duration of CIDR (5 to 8 days exposure) did not significantly affect pregnancy rates in recipient cows [45]. The induction of multiple ovulations using eCG is another potential approach to increasing circulating progesterone concentrations [84]. Nasser, Reis, Oliveira, Bo and Baruselli [85] administered a CIDR and injection of oestradiol benzoate to 304 beef heifers, either with or without an injection of progesterone (50 mg), to synchronise recipient cows for embryo transfer. The heifers treated with eCG at Day 5 had more CLs and a higher plasma progesterone concentration which, in turn, tended to increase pregnancy rate [85]. Similarly, treating ewes with eCG on Day 10 post insemination had a marginal positive effect on pregnancy establishment [35].

Further research into the more effective use of CIDRs between ovulation and the establishment of maternal recognition of pregnancy is required, as progesterone is crucial for the maternal support of the conceptus [86]. Supplementation of progesterone in lactating dairy cows following ovulation did not compromise CL volume or subsequent circulating progesterone. However, in dairy cows, supplementation did not improve AI pregnancy rates, and decreased pregnancy rates per transfer for embryo recipients [87]. The timing of progesterone supplementation is critical for embryo and CL development [88]. A review by Lonergan and colleagues summarised the key timings of progesterone supplementation and the subsequent effects on reproductive outcomes [82], and suggested that the type of animal (lactating or dry), as well as endogenous progesterone concentrations within the animal, also affect the outcomes. As such, further research into the timing of supplementation for recipient animals within specific protocols is warranted.

Assessment of recipient CL size and progesterone concentration prior to synchronisation would enable recipients to be placed within an intervention protocol that would suit their reproductive requirements. A recipient that has lower progesterone concentrations may be more suited to treatment with a post-transfer CIDR until maternal recognition of pregnancy is established, in comparison to a recipient who has an adequate progesterone concentration and hence may be more suited to the protocol of one CIDR prior to ET. Consequently, establishing the recipient’s ovarian function prior to synchronisation is paramount in maximising the recipient’s ability to carry the embryo to full term, based on how efficient the synchronisation protocol is.

## 6. Seminal Plasma

Seminal plasma is a complex fluid comprised of secretions from the seminal vesicles, the prostate, bulbourethral glands and from the seminiferous tubule lumen [89]. Seminal fluid is rich in simple sugars, buffers, antioxidants, hormones and various proteins that facilitate sperm survival and transport through the female reproductive tract [90]. Crucial to the viability and integrity of sperm, seminal plasma also balances embryotrophic and embryotoxic signals within the female’s reproductive tract [91]. Seminal plasma proteins interact with the vaginal, cervical and uterine epithelium to induce changes in the immune responsiveness of the female to sperm and the developing embryo [92]. Exposure of the cervix and uterus to seminal fluid elicits dramatic changes in cytokine expression and leucocyte populations. In vivo experiments studying the effects of seminal plasma on the female reproductive tract and embryo–uterine development have not been conducted in ruminants, but have been undertaken in mice [93], humans [94], and pigs [95]. In ruminants, recent work has determined that pregnancy at Day 32 and 60, but not overall calving rate, was affected by the use of seminal plasma at time of AI [96]. However, the overall fertility in this herd was high, perhaps indicating that seminal plasma may be best used for cattle herds with low fertility.

The insemination response is hypothesised to effect four main categories that are important for early embryogenesis and effective implantation; (1) clearance of superfluous sperm and microorganisms, (2) activation of female immune responses specific to paternal transplantation proteins, (3) tissue remodelling associated with endometrial receptivity and (4) activation of cytokines and growth factors implicated in pre-implantation embryogenesis [97]. Furthermore, inhibiting the potentially harmful immune activity to paternal major histocompatibility complex antigens associated with the conceptus may facilitate implantation and placental development [98].

Absence of seminal plasma leads to the downregulation of embryo trophic factors and the upregulation of embryo toxic factors, leading to an increased chance of pregnancy termination [91]. TGF-β and E-series PGF are synthesised by seminal vesicles and play a crucial role in inducing the female reproductive tract to synthesise cytokines and chemokines which influence the immune response of the recipient to enable tolerance to the embryo [98]. Whilst pregnancy can be established without the exposure of a female to seminal plasma, the full benefit of seminal plasma is not known and requires further research [91]. Robertson, Bromfield, Glynn, Sharkey and Jasper [97] summarised evidence from multiple species that indicated success rate and quality of pregnancy can clearly be compromised if females are not exposed to seminal plasma. Little research has been conducted in sheep or cattle determining the effect of seminal plasma-induced endometrial inflammation to support pregnancy establishment [99]. Small improvements to reproductive performance are likely, as a mechanistic role of seminal plasma in rodents appears to influence the reproductive tract at a cellular and molecular level [99]. Whilst seminal plasma is not necessary for a successful pregnancy, marked improvements in developmental programming may exist, with the potential to improve growth and productivity [99]. Therefore, rather than seminal plasma being considered as a sperm transport medium, it needs to be acknowledged as a means of communication between the female and male reproductive tracts, and if included in ART protocols may have the potential to increase ET success rates by preparing the tract for the transferred embryo.

## 7. Conclusions

With the current state of the Australian livestock industry, new approaches need to be implemented to improve current reproductive rates. The use of assisted reproductive technologies is increasing within the Australian sheep and cattle industries; however, widespread adoption is unlikely with the current variability and high financial costs. There is substantial variability seen in techniques used as intervention strategies for ARTs, individual animal responses to ARTs as well as reproductive outcomes. We have established that poor reproductive outcomes are often the result of early embryo losses, as a large portion of reproductive wastage was observed prior to day 30 of gestation. With this in mind, whilst the current literature is assessing the selection of animals to use as donors for ART’s, little work has assessed the selection of recipient animals. Recipient animals include all animals expected to establish and maintain pregnancy following some form of reproductive intervention (i.e., natural mating, AI or embryo transfer). A universal method of selection would reduce animal variability and therefore increase the outcome from ARTs. The use of genetic selection alone is not sufficient, as the environment and management of the animals plays a crucial role in reproductive outcomes. As such, a phenotypic marker of selection is a novel way forward, as it can be implemented across all avenues of ARTs, understanding the development and control of ovarian function enables a marker reflective of ovarian activity to be utilised. Our review proposes that anti-Müllerian hormone is a possible ovarian marker. The possibility of selecting animals based on circulating AMH levels, together with adequate nutritional management for body condition and the possibility of early uterine priming to increase embryo receptivity, may reduce the variability seen in Australian livestock reproductive outcomes.
